# Computational Exploration of Single-Nucleotide Polymorphisms in the Human hRAS Gene: Implications and Insights

**DOI:** 10.7759/cureus.53119

**Published:** 2024-01-28

**Authors:** Sankar Dakshitha, Boopathi Priya dharshini, Vasugi Suresh, Elangovan Dilipan

**Affiliations:** 1 Physiology, Saveetha Dental College and Hospitals, Saveetha Institute of Medical and Technical Sciences, Saveetha University, Chennai, IND

**Keywords:** single-nucleotide polymorphism (snp), bioinformatic tools, amino acid, hras gene, computational biology

## Abstract

Background

A group of genes called oncogenes includes the Harvey rat sarcoma virus (*hRAS*) gene. Along with hRAS, Kirsten rat sarcoma viral oncogene homolog (*kRAS*) and neuroblastoma RAS viral oncogene homolog (*nRAS*) genes belong to the Rat sarcoma (Ras) family of oncogenes. These three genes result in Rho guanosine triphosphate hydrolases (GTPases) as their protein product. Instructions for producing the protein hRAS, which is mainly involved in controlling cell division, are provided by the *hRAS *gene. The hRAS protein transfers signals from outside through a process called signal transduction. Because the hRAS protein is a GTPase, it changes the chemical guanosine-5'-triphosphate (GTP) into guanosine diphosphate (GDP). GTP and GDP molecules operate as switches to turn on and off the hRAS. This study aimed to anticipate the structure and stability of the protein resulting from missense single-nucleotide polymorphisms (SNPs) in the human *hRAS* genes.

Methodology

To investigate the possible negative effects associated with these SNPs, bioinformatic analysis is typically essential. The following tools were employed for forecasting harmful SNPs: Scale-Invariant Feature Transform (SIFT), Protein Analysis Through Evolutionary Relationships (PANTHER), non-synonymous SNP by Protein Variation Effect Analyzer (PROVEAN), and non-synonymous SNP by Single Nucleotide Polymorphism Annotation Platform (SNAP).

Results

The present study identified a total of 11 SNPs using the SIFT approach, which were shown to have functional significance. Only two of these 11 SNPs were determined to be tolerable, whereas nine were shown to be detrimental. Among the 11 SNPs analyzed, seven (Q61H, Q99H, K117R, A121D, A146V, R169W, R169Q) were classified as *possibly damaging*,and four (G13V, Q22K, Q61K, Q13V) were categorized as *probably benign* according to the predictions made by PANTHER tools. Therefore, the seven SNPs were identified as high-risk SNPs.

Conclusions

Given that SNPs have the potential to be candidates for cellular alterations brought on by mutations that are associated with cancer, this study provides vital information about how SNPs might be utilized as a diagnostic marker for cancer.

## Introduction

The human Harvey rat sarcoma virus (*hRAS*) gene, which is found on chromosome 11p15.5, controls the cellular signaling route through a molecular switch mechanism, which causes cell division. The RAS protein, which is frequently imbalanced in human cancer, encodes *hRAS*. Mutations in *hRAS *have been documented in cases of thyroid cancer, salivary gland cancer, bladder and mouth cancer, and rhabdomyosarcoma [1]. The bulk of genetic diversity in the human population is caused by single-nucleotide polymorphisms (SNPs), which are described as single-base variations in a DNA fragment. Synonymous SNPs in the protein-coding regions can be categorized into groups based on how the polymorphism affects the amino acid (those that do not alter the amino acid), non-synonymous SNPs (nsSNPs), which alter the amino acid, and nonsense mutations, in which the SNP causes a stop codon. nsSNPs frequently have negative impacts on protein structure or function, even though many SNPs are phenotypically neutral. Protein coding areas contain nsSNPs that cause an amino acid substitution in the associated protein product. Because of this, nsSNPs are frequently linked to human disease and have the power to change the stability, structure, or function of proteins. According to earlier research, nsSNPs are responsible for almost half of the mutations linked to inherited genetic illnesses [2,3].

*RAS *genes, particularly those in *hRAS*, are among the most frequently found mutations in human malignancies [4]. *RAS *genes are small Rho guanosine triphosphate hydrolases (GTPases) that play a major role in regulating numerous signaling pathways that are connected to a variety of biological processes [5,6]. In the human genome, SNPs are the most prevalent genetic variants. They entail changing just one nucleotide at a particular location in the DNA sequence. Because SNPs can alter gene activity, protein structure, and overall phenotype, they are crucial targets for research into drug response, disease susceptibility, and personalized treatment. The small GTPase protein encoded by the human *hRAS *gene is a proto-oncogene that participates in cell signaling pathways. Mutations in the *hRAS *gene have been connected to a variety of diseases, such as cancer and developmental issues. Determining the SNPs in the *hRAS *gene impact illness etiology and patient classification requires an understanding of the possible functional implications of these variants. In silico analysis has changed the field of genomics research by using computational approaches [7]. A range of approaches have also been used to review the SNPs. Among these are oligonucleotide ligation, primer extension, direct sequencing, nuclease cleavage of mismatches, and oligonucleotide hybridization [8,9].

SNPs account for more than 90% of all differences in human nucleic acid sequences. Amino acid substitution is caused by nsSNPs, which can alter protein function and produce pathogenic symptoms [10]. SNPs are differences in a single nucleotide that exist in the genome at a frequency greater than 1%. SNPs are found in roughly every 3,000 base pairs of the human genome and the frequency at which the various alleles arise varies throughout populations [11,12]. Genes that regulate cell proliferation may become downregulated as a result of nsSNPs, leading to uncontrollably dividing cells, the growth of cancer cells, and a variety of other human diseases. Furthermore, it has been documented that the investigation of functional SNPs in cancer genes improves human health [13]. Further, Ras family members with GTPase activity include the *Ras *genes. Ras guanosine-5'-triphosphate (GTP) and Ras guanosine diphosphate (GDP) serve as the active and inactive states, respectively, of these genes, which act as molecular switches in the cell [14]. Numerous researchers have been interested in the role Ras proteins play in the regulation of cellular signaling pathways during the past few decades [15]. According to studies, these proteins play important functions in controlling cell death, regulating cell motility, and planning cell proliferation [16]. A variety of post-translational modifications (PTMs) at the C-termini of each protein localize each RAS member’s specialized function to a separate subcellular membrane, where it then activates a particular signaling pathway [15]. Plus, the *hRAS *gene produces a GTPase known as the hRAS protein, which serves as a crucial molecular switch for connecting extracellular signals to intracellular pathways involved in cell growth and differentiation. Given the *hRAS *gene’s crucial function in controlling cellular responses, genetic variants, in particular, SNPs, may disrupt regular signaling cascades and lead to a variety of disease phenotypes [17].

The functional effects of SNPs in the *hRAS *gene must, therefore, be thoroughly investigated to understand their possible roles in illness etiology, therapy responsiveness, and overall patient outcomes [1]. Several computational techniques have been developed to in silico forecast the deleteriousness of nsSNPs due to the potential functional implications of these genetic variations. Scale-Invariant Feature Transform (SIFT) is one of these techniques [18]. This study examined the stability, function, and protein structure of SNPs known to alter the amino acid sequences of the genes. Bioinformatics technologies such as Protein Analysis Through Evolutionary Relationships (PANTHER) were utilized to research gene prediction, sequence alignment, and protein structure prediction [19] To estimate how an amino acid substitution would affect a protein’s biological function, the Protein Variation Effect Analyzer (PROVEAN) was utilized [20]. The present study aims to perform a comprehensive in silico analysis of SNPs in the *hRAS *gene to identify potential genetic variations and to explain their impact on protein structures and functions.

## Materials and methods

Retrieval of variant datasets

Online Mendelian Inheritance in Man and Ensemble was used to acquire information about the human hRAS gene. The Kyoto Encyclopaedia of Genes and Genomes (KEGG) provided the protein sequence after the SNP data were collected from the National Centre for Biotechnology Information (NCBI) dbSNP (database of SNP). The known SNP variants identified in *hRAS *gene and implicated in cancer are presented in Table 1.

**Table 1:** List of diseases caused by single-nucleotide variant in hRAS gene retrieved from the UniProt database.

dbSNP ID	Amino acid change	Disease
rs104894230	G>V	Bladder carcinoma
rs104894228	G>R	Somatic mutation
rs104894226	G>D	Costello syndrome
rs121917758	T>I	Costello syndrome
rs104894227	K>R	Costello syndrome
rs104894231	A>T	Costello syndrome
rs121917759	A>V	Costello syndrome
rs104894229	G>S	Oral cancer
rs121917757	Q>K	Germline mutation
rs121917756	E>K	Germline mutation
rs28933406	Q>K	Thyroid cancer

In this study, 38 SNPs were retrieved from dbSNP (Table 2) and evaluated the SNPs using bioinformatic tools.

**Table 2:** List of SNP IDs of hRAS gene retrieved from the database of single-nucleotide polymorphism.

dbSNP ID	Alleles	Clinical significance
rs104894226	C>A,G,T	Likely pathogenic
rs121917757	G>A,T	Likely pathogenic
rs28933406	G>A,C,T	Likely pathogenic
rs121913496	C>A,G,T	Likely pathogenic
rs199656012	C>A,G,T	Uncertain
rs104894227	T>A,C	Pathogenic
rs376667492	G>A,C,T	Uncertain
rs121917759	G>A,C,T	Likely pathogenic
rs151229168	G>A,C	Uncertain
rs142218590	C>A,T	Uncertain
rs878854761	T>A,C	Uncertain
rs1447218022	G>A,C	Uncertain
rs764622691	T>C,G	Uncertain
rs1554885164	A>C,G,T	Uncertain
rs763376142	G>A,C	Likely benign
rs727503094	GC>AA,AG,AT,TA,TT	Likely pathogenic
rs104894230	C>A,G,T	Likely pathogenic
rs104894229	C>A,G,T	Likely pathogenic
rs104894228	C>A,G,T	Likely pathogenic
rs1589793707	A>C,T	Uncertain
rs1554885139	C>A,G,T	Uncertain
rs1445047175	T>C	
rs1226305644	C>A,G,T	Uncertain
rs1851315835	C>A,G,T	Uncertain
rs1348427922	G>A,T	Uncertain
rs775056058	A>G,T	Uncertain
rs1060502663	T>A,C	Uncertain
rs398122809	->TCT	Pathogenic
rs750680771	C>A,G,T	
rs1028352031	A>T	Uncertain
rs1564789708	C>A,G,T	Uncertain
rs1851286467	A>C,G,T	Likely benign
rs1564789700	A>G,T	Likely pathogenic
rs1277340795	C>A,G,T	Likely pathogenic
rs727503093	C>A,G,T	Likely pathogenic
rs730880460	C>A,G,T	Pathogenic
rs1851251123	G>A,C,T	Uncertain
rs756367459	A>G	Uncertain

Deleterious SNP prediction by SIFT

SIFT predicts permitted and harmful substitutions for every query sequence location using multiple alignment information [21]. This procedure involves searching for similar protein sequences, selecting closely related sequences with similar functions, obtaining multiple alignments, and calculating normalized probabilities for all possible substitutions at each position. Normalized probabilities below 0.05 suggest intolerant or harmful substitutions at each site, and those over 0.05 are projected to be tolerated [22].

Evolutionary relationships by PANTHER

PANTHER is available at http://www.pantherdb.org/tools/. It employs statistical modeling based on the Hidden Markov Model (HMM) and evolutionary connections [23]. The user has provided a query that includes a protein sequence for the hRAS protein (P01112) as well as an amino acid substitution at a specific place for the organism *Homo sapiens*.

Prediction of the functional effect of non-synonymous SNP by SNAP

The Screening of Non-acceptable Polymorphism 2 (SNAP2) tool may be accessed for free at https://www.rostlab.org/services/snap/. Its aims to detect functional impacts caused by mutations. The hRAS protein sequence (P01112) was the input query that was submitted. The neutral network classification approach as the basis for this methodology [24].

## Results

Retrieval of variant datasets

The dbSNP database contains both verified and unverified polymorphisms. Despite this limitation, we selected to use the dbSNP due to its comprehensive collection of allelic frequencies for most nsSNPs of *hRAS*. It is considered the most thorough database for SNPs. A total of 50 SNPs were obtained from the dbSNP database. A total of 11 SNPs were isolated, with nine identified as detrimental and just two classified as tolerated.

Deleterious SNP prediction by SIFT

The SIFT method is used to determine if nsSNPs are deleterious or tolerated. The SIFT technology plays a crucial role in identifying potential disease candidates from missense variations. Amino acid substitutions with a normalized probability cutoff value of b0.05 were anticipated to be detrimental, whereas those with a probability of ≥0.05 were expected to be tolerated. SIFT tools were used to predict and identify the deleterious and tolerated SNPs. Out of 11 SNPs, nine (G13V, G13D, Q22K, Q61K, Q61H, Q99H, K117R, A146V, R169W) were predicted as *harmful* and two (A121D, R169Q) were tolerated by SIFT tools. Hence, those nine SNPs were high-risk SNPs (Table 3).

**Table 3:** List of non-synonymous single-nucleotide polymorphisms found to be functionally significant using the Scale-Invariant Feature Transform tool.

rsID	Amino acid change	SIFT	Score
rs104894226	G13V	Deleterious	0.021
rs104894226	G13D	Deleterious	0.023
rs121917757	Q22K	Deleterious	0.001
rs28933406	Q61K	Deleterious	0.016
rs121913496	Q61H	Deleterious	0.002
rs199656012	Q99H	Deleterious	0.049
rs104894227	K117R	Deleterious	0.001
rs376667492	A121D	Tolerated	0.201
rs121917759	A146V	Deleterious	0
rs151229168	R169W	Deleterious	0.002
rs142218590	R169Q	Tolerated	0.946

Evolutionary relationships by PANTHER

PANTHER provides predictions for three potential outcomes, namely, *probably damaging* (when time is more than 450 million years), *possibly damaging* (when time is between 450 million years and 200 million years), and *probably benign* (when time is less than 200 million years). Location-specific evolutionary preservation is quantified as the length of time (in millions of years) that a location in a current protein has remained unchanged, tracing back to its reconstructed direct predecessors. If the posture is maintained for a longer time, it is more probable to have adverse consequences. PANTHER tools were used for gene prediction, sequence alignment, and protein structure prediction. Out of 11 SNPs, seven (Q61H, Q99H, K117R, A121D, A146V, R169W, R169Q) were predicted as *possibly damaging*, and four (G13V, Q22K, Q61K, Q13V) were identified as *probably benign* by PANTHER tools. Hence, these seven SNPs were high-risk SNPs (Table 4).

**Table 4:** List of single-nucleotide polymorphisms predicted to be damaging according to PANTHER.

Substitution	Preservation time	Message	Pdel
G13V	15	Probably benign	0.01
G13D	192	Probably benign	0.31
Q22K	75	Probably benign	0.17
Q61K	183	Probably benign	0.23
Q61H	36	Possibly damaging	0.5
Q99H	570	Possibly damaging	0.5
K117R	261	Possibly damaging	0.5
A121D	115	Possibly damaging	0.5
A146V	174	Possibly damaging	0.5
R169W	156	Possibly damaging	0.5
R169Q	127	Possibly damaging	0.5

Prediction of the functional effect of non-synonymous SNP by SNAP

**Figure 1:**
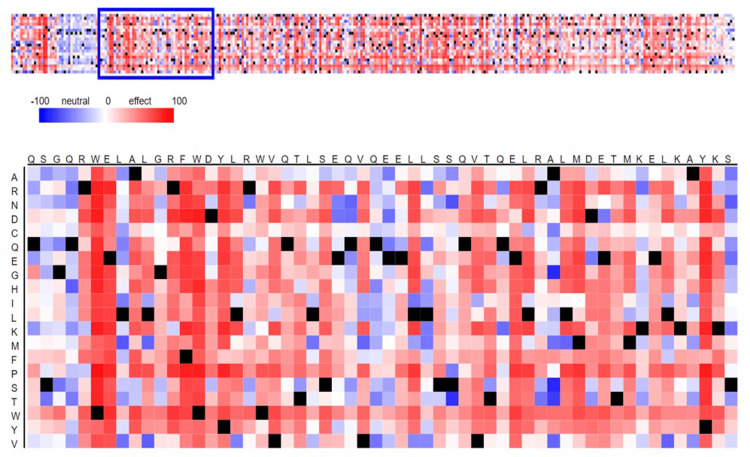
Predicting functional effects of sequence variants of Harvey rat sarcoma virus gene by Single Nucleotide Polymorphism Annotation Platform tool.

The matrix, organized by color coding, illustrates the anticipated impact of amino acid substitutions on the protein’s activity. In general, the color red is used to represent an anticipated harmful or negative impact, whereas blue is used to represent a projected neutral effect (Figure 1). The intensity of the color may correspond to the level of confidence in the forecast. The sequence of amino acids in a segment of the hRAS protein, where each location corresponds to a column in the matrix. The left axis has 20 standard amino acids, each indicating a potential replacement at each location. SNAP2 often offers a level of confidence in its forecasts, which may be shown by the intensity of the colors. The present results indicate that some amino acid changes are anticipated to have a more pronounced detrimental impact (shown by deeper red dots), while others are expected to be neutral (indicated by blue cells). These predictions might help in planning more experiments to determine how these mutations affect the function of the hRAS protein, which is very important in many types of cancer.

## Discussion

The SNPs of the human *RAS *gene were collected from the NCBI dbSNP database. The retrieval and subsequent analysis of variant databases, particularly SNPs, using SIFT, PANTHER, and SNAP tools have provided valuable insights into the potential functional impact of genetic variations. The *hRAS* gene variations have been linked to several illnesses, including cancer and developmental disorders. Our findings provide the basis for future investigations into any possible relationships between particular SNPs and disease risk [25]. The utilization of SIFT tools enabled the prediction of deleterious and tolerated SNPs within the dataset extracted from the dbSNP database. The majority of SNPs (nine out of 11) were classified as *harmful* by SIFT, indicating a high proportion of potentially damaging variants. Notably, the specific identification of these harmful SNPs, such as G13V, G13D, Q22K, Q61K, Q61H, Q99H, K117R, A146V, and R169W, suggests a significant potential risk associated with these genetic variations. The identification of high-risk SNPs by SIFT highlights specific genetic variants that may have a notable impact on protein function. These harmful SNPs may be associated with increased susceptibility to diseases or altered biological processes, warranting further investigation into their potential roles in health and disease. All SNPs had been sent for further analysis. Among the nine deleterious SNPs, four had low Pdel scores (0.01, 0.31, 0.17, and 0.23), and were probably benign, which were confirmed to become cancerous cells. The other five SNPs, with a score of 0.5, were all probably damaging.

Two of the SNPs, shown to be tolerated in the SIFT tool, were revealed to be probably damaging according to the PANTHER tool. The 125 nsSNPs of the HNPCC genes were identified from a total of 1,970 SNPs, which were subjected to the SIFT and PolyPhen algorithms. SIFT, a method for separating intolerant from tolerant variants, categorized 22 out of 125 variants (18%) as *intolerant*. Forty out of 125 amino acid changes (32%) were classified as *probably or possibly damaging* by Polymorphism Phenotyping (PolyPhen) [26]. Further study into the population-specific impact of these SNPs on health risk and treatment response is essential given the diversity of human populations [27]. Additional research verified the functional effects of the *hRAS *gene SNPs that Uniprot had previously evaluated. The harmful SNPs in this investigation were predicted using Uniprot. The database was searched to see if each SNP changed an amino acid that was known to be crucial for the protein’s structure or function. Based on these results, most SNPs were predicted to have deleterious consequences on the protein’s structure and function, some were shown to be benign, some were predicted to be pathogenic or likely pathogenic, and a few had uncertain phenotypes. The present research lays the groundwork for focused experimental studies to confirm the effects of certain SNPs detected in the *hRAS *gene. Furthermore, a study conducted in 2003 collected DNA from fresh tumor tissue of patients who were confirmed for urinary bladder cancer to screen for mutation in the coding region of the human *RAS *gene using a DNA sequence analysis. This study concluded that the H-ras proto-oncogene’s common genetic variation appears to be epidemiologically significant for bladder cancer risk and development [28]. PANTHER tools were employed to assess evolutionary relationships, gene prediction, sequence alignment, and protein structure prediction. The analysis categorized a subset of SNPs as *possibly damaging*. Out of 22 SNPs, seven (Q61H, Q99H, K117R, A121D, A146V, R169W, R169Q) SNPs were predicted by PANTHER tools. The validity of high-risk SNP identification is increased by the agreement between two independent prediction methods, boosting confidence in the accuracy of the findings. Furthermore, the majority of pathogenic nsSNPs, regardless of whether they increase or reduce protein stability, occupy conserved amino acid sites, according to evolutionary conservation studies. In the end, the high-risk nsSNPs were determined to be those that significantly reduced the stability of proteins. Combining these data, we discovered that the TP53 V216G and L194H variants, the ATM V2865T and V2906A variants, and the BRCA1 V1687G and V1736G variants were all extremely harmful mutations that significantly reduce protein stability and may change the activities of the corresponding proteins [13].

Functional effects of sequence variants of the *hRAS* gene by SNAP2 screening predict the functional significance of changes in the *PIK3CA *gene sequence. The neutral effect of the gene is represented by the blue sections, and its negative effects are represented in red, Except for one patch that has more blue pixels than the rest (Figure 2), the majority of the strip is covered with red pixels, indicating that it is harmful and dangerous. The in silico analysis predicted a range of possible impacts for the discovered SNPs. These included alterations to protein coding sequences, expected disruptions of protein-protein interaction sites, along with modifications to regulatory regions. The dbSNP database revealed a dataset of SNPs in the human *JAK1 *gene (gene ID: 3716). The human *JAK1 *gene contains a total of 52,315 SNPs, of which 1,957 have been observed to occur in the exon region. Only 0.92% of all SNPs known to exist in the human *JAK1 *gene are non-synonymous, of which 483 (473 missense and 10 nonsense) were found. The harmful nsSNPs were located using a variety of computational techniques, including SIFT, SNAP2, Polyphen-2, PROVEAN, and PANTHER. Functionally important nsSNPs were found after a preliminary screening process using SIFT. According to SIFT, missense variants are either harmful (less than 0.05) or tolerated (equal to 0.05). Among 483 nsSNPs, SIFT found 20 alterations to be harmful and 30 to be acceptable [29]. In addition, a study conducted by Sathyan et al. in 2005 evaluated the influence of SNPs in *hRAS *genes in oral cancer susceptibility. The research examined the link between H-Ras (C81T) and cyclin D1 (A870G and C1722G) gene SNPs and oral cancer risk in 176 oral cancer cases and 142 hospital-based controls matched for age and sex. The variant C allele of the H-Ras C81T polymorphism may be a low-penetrating genetic modifier for oral carcinogenesis, and men were more likely to play this function [30]. The research employed in-silico methods to anticipate how rare genetic mutations may alter HRAS protein function. The 50 nsSNPs were discovered, 23 of which were projected to be deleterious or damaging in the HRAS gene exon. The HRAS protein’s G60V, G60D, and D38H mutants have greater binding energies than the wild-type protein, which may affect oncogenic signaling cascades and malignancies. The entire research analysis sheds light on the potential role of *hRAS *gene nsSNPs in cancer [4]. One of the limitations of in silico analysis is the difficulty of accurately predicting the context-dependent effects of SNPs. Cellular responses could vary based on the kind of tissue, developmental stage, and external circumstances. The real impacts of SNPs in the hRAS gene might be more complex and wide-ranging, even though our projections provide some insight into possible effects. Experimental data from several biological contexts must be combined to completely understand the spectrum of SNP effects.

Limitations

The capacity of computational models to properly reflect the complexity of biological systems is limited, and this constraint comes with certain limits. The algorithms and databases that were used are directly responsible for the correctness of the findings. Analyses performed in silico are theoretical and need confirmation via experimentation. One of the major limitations of the research is that it did not conduct laboratory studies to validate the computed predictions. For SNP information, the research makes use of databases that are already in existence. When these databases are either missing information or include mistakes, it may have a negative impact on the comprehensiveness and accuracy of the process. If the SNP data that were utilized was skewed toward certain ethnicities or groups, it is possible that the conclusions cannot be generalized to other population groups. In the field of genetic research, this is a typical problem.

## Conclusions

The analysis of SNPs in the human hRAS gene using in silico analysis has provided valuable insights into the potential functional implications of genetic variations in this crucial signaling pathway. Using an array of bioinformatics tools, databases, and prediction algorithms, we investigated the possible impact of SNPs on protein structure, function, and regulation. Many of the SNPs found in this in silico study were predicted to be harmful, implying that they could be potential candidates for cancer-related mutations that lead to changes in cells. Many SNPs have an impact on the *hRAS *gene. We currently know that SNPs can be employed as a diagnostic marker in cancer research.
